# Decreased respiratory system compliance on the sixth day of mechanical ventilation is a predictor of death in patients with established acute lung injury

**DOI:** 10.1186/1465-9921-12-52

**Published:** 2011-04-22

**Authors:** Eric J Seeley, Daniel F McAuley, Mark Eisner, Michael Miletin, HanJing Zhuo, Michael A Matthay, Richard H Kallet

**Affiliations:** 1 Departments of Medicine and Anesthesia, Cardiovascular Research Institute, University of California, San Francisco, San Francisco California, USA; 2 Respiratory Medicine Research Programme, Centre for Infection and Immunity, Queen's University of Belfast, Belfast Northern Ireland; 3 Department of Medicine, William Osler Health Centre, Toronto, Canada; 4 Department of Anesthesia, University of California, San Francisco at San Francisco General Hospital, San Francisco California, USA

## Abstract

**Background:**

Multiple studies have identified single variables or composite scores that help risk stratify patients at the time of acute lung injury (ALI) diagnosis. However, few studies have addressed the important question of how changes in pulmonary physiologic variables might predict mortality in patients during the subacute or chronic phases of ALI. We studied pulmonary physiologic variables, including respiratory system compliance, P/F ratio and oxygenation index, in a cohort of patients with ALI who survived more than 6 days of mechanical ventilation to see if changes in these variables were predictive of death and whether they are informative about the pathophysiology of subacute ALI.

**Methods:**

Ninety-three patients with ALI who were mechanically ventilated for more than 6 days were enrolled in this prospective cohort study. Patients were enrolled at two medical centers in the US, a county hospital and a large academic center. Bivariate analyses were used to identify pulmonary physiologic predictors of death during the first 6 days of mechanical ventilation. Predictors on day 1, day 6 and the changes between day 1 and day 6 were compared in a multivariate logistic regression model.

**Results:**

The overall mortality was 35%. In multivariate analysis, the PaO_2_/FiO_2 _(OR 2.09, p < 0.04) and respiratory system compliance (OR 3.61, p < 0.01) were predictive of death on the 6^th ^day of acute lung injury. In addition, a decrease in respiratory system compliance between days 1 and days 6 (OR 2.14, p < 0.01) was independently associated with mortality.

**Conclusions:**

A low respiratory system compliance on day 6 or a decrease in the respiratory system compliance between the 1^st ^and 6^th ^day of mechanical ventilation were associated with increased mortality in multivariate analysis of this cohort of patients with ALI. We suggest that decreased respiratory system compliance may identify a subset of patients who have persistent pulmonary edema, atelectasis or the fibroproliferative sequelae of ALI and thus are less likely to survive their hospitalization.

## Background

Acute lung injury (ALI) is a major cause of morbidity and mortality in ICUs throughout the world [1-3]. Despite improvements in ventilation strategies and supportive care, the mortality from ALI remains between 30-60%. Due to this high mortality, rescue therapies such as extracorporeal membrane oxygenation (ECMO), inhaled nitric oxide, prone positioning and high frequency oscillatory ventilation are often considered for patients who are perceived to be at the highest risk of death.

Identifying patients at the highest risk of death has been a barrier to effectively testing and implementing these therapies. Physiologic measures that predict increased mortality when measured at the time of admission include an elevated dead space fraction, a low oxygenation index and an increased extravascular lung water [3-5]. However, important decisions regarding therapeutic interventions and changes in goals of care are often made later in the course of illness. Few studies have focused on pulmonary physiologic variables that might be associated with death during this crucial time period. Thus, we performed a study of pulmonary physiologic variables in a cohort of patients who survived more than 6 days of mechanical ventilation. The objectives of this study were two-fold. First, using multivariate analysis, we hoped to identify independent predictors of death that might help identify patients at risk for poor outcomes and thus best suited for experimental therapies for ALI. Second, through a physiologic investigation of pulmonary mechanics over the first 6 days of mechanical ventilation, we sought to develop a deeper understanding of the pulmonary pathophysiology that might lead to death during ALI.

## Material and methods

### Subjects

Patients who met the North American-European consensus conference definition of ALI [6] in both medical and surgical intensive care units were identified prospectively as a part of ongoing clinical trials of ALI between July 1^st ^2002 and June 30^th ^2003. All enrolled patients were 18 years or older and there were no exclusion criteria for enrollment. Select data on 149 consecutive patients were collected prospectively, and additional data were then extracted from the medical record retrospectively. In order to identify predictors of mortality in established ALI, 93 out of 149 patients who survived more than 6 days of mechanical ventilation for ALI were included in this study. The 56 (149 minus 93) patients excluded from the original cohort were either extubated (27 patients) or died (29 patients) before the 6^th ^day of mechanical ventilation. The association between several pulmonary physiologic variables measured at the time of study enrollment (for all 149 patients) and the outcome of death has been published [4]. This study was conducted at the University of California Moffitt-Long Hospital, a tertiary university referral center, and at San Francisco General Hospital, a large, inner-city hospital and Level 1 trauma center. Data collection was approved by the institutional review board of the University of California, San Francisco. For data collected retrospectively, the requirement for written informed consent was waived. Ventilator management was at the discretion of the critical care team. However, both hospitals had implemented the lung-protective ventilation protocol of the ARDS Net trial on either a formal or an informal basis and >90% of patients were noted to be on either volume controlled or pressured regulated volume controlled ventilatory modes.

### Data Collection

The plans for data collection and analyses were defined prospectively, before review of the medical records began. Data were recorded at a daily reference time between 0600 and 1000. A reference quasi-static respiratory system compliance (Crs) reflected the average daily Crs in a subset of subjects [7]. The quasistatic respiratory compliance was calculated by dividing the difference between the tidal volume and the volume compressed in the ventilator circuit by the difference between the plateau pressure and the positive end-expiratory pressure or [(TV - Volume left in circuit)/(Ppl - PEEP)].

Clinical data were abstracted from the medical record for up to 6 days or until the time of death or extubation, whichever occurred first. These data included the etiology of ALI, coexisting medical illnesses, the use of glucocorticoids or other causes of immunosuppression, fluid intake/output and balance, vital signs, and chest radiographic findings. The clinical disorder associated with ALI was considered primary if the cause was pneumonia, aspiration, direct lung trauma, or inhalational injury. All other causes were considered secondary.

Laboratory data collected were electrolytes, blood urea nitrogen, creatinine, white blood cell count, and hematocrit. Mechanical ventilation variables included arterial blood gases, peak inspiratory pressure, plateau pressure (P_plat_), positive end-expiratory pressure (PEEP), mean airway pressure (mean Paw), tidal volume (V_T_) in both mL and mL/kg predicted body weight (PBW), respiratory frequency (f), and minute ventilation (). Calculated variables included the lung injury score (LIS) [8], APACHE II [9], SAPS II [10], PaO_2_/FiO_2_, and respiratory system compliance. The oxygenation index (OI) was calculated as [mean airway pressure x FiO_2 _x 100] ÷ PaO_2 _[11]. For patients with trauma-induced ALI, the Injury Severity Score [12] was determined.

### Statistical Analysis

The primary outcome variable of this study was death prior to hospital discharge. Multiple physiologic variables on each day of mechanical ventilation were compared using bivariate analyses. These bivariate analyses were considered exploratory and undertaken to identify variables for the multivariate analysis, thus, p values were calculated without correction for multiple comparisons. Continuous normally distributed variables were compared using a Student's t-test, and categorical variables were compared using the Fisher's exact test. Select predictor variables that were statistically significant or that were of *a priori *clinical interest were entered into a backward stepwise, multivariate logistic regression model. Separate multivariate logistic regression models were developed for predictor variables measured at day 1, day 6, and the change in value between day 1 and 6. Stata 9.0 (StataCorp, College Station, Texas) computer software was used for statistical analysis. All interval data in tables and text are presented as mean with standard deviation in parentheses. Data presented in graphs are mean with error bars indicating the standard error of the mean (SEM). The odds ratios for death are calculated per standard deviation increase or decrease in each variable to allow for equal comparisons between different variables. The goodness-of-fit of the logistic-regression model was assessed with the Hosmer-Lemeshow test, all p values for the H-L test were >0.05, indicating that the model was well calibrated. Results were considered to be statistically significant at two-tailed p < 0.05.

## Results

### Cohort Characteristics

The mortality of this group of 93 patients who survived 6 days of lung-protective ventilation for ALI was 35% (95% CI: 26%-46%). The observed mortality was higher than the mortality estimated by the SAPS II (27%) or APACHE II (30%) score on the first day of ALI. ALI was due to a variety of primary (38/93, 41%) and secondary (55/93, 59%) causes, including pneumonia (24/93, 26%), sepsis (20/93, 22%) and aspiration (5/93, 5%). Nearly a quarter of the patients were immunosuppressed due to HIV, malignancy or organ transplantation. In addition, a substantial fraction had cirrhosis. This cohort of patients had moderate lung injury with an average PaO_2_/FiO_2 _of 147 ± 60 cm H_2_O, OI of 11.4 ± 8 cm H_2_O/mm Hg and initial respiratory system compliance of 28 ± 10 ml/cm H_2_O (Table 1). The average duration of mechanical ventilation for the entire cohort was 21.4 ± 25.1 days. There was no difference in duration of mechanical ventilation between survivors and non-survivors (Table 1). The initial tidal volume in this cohort of patients was 7.4 ml/kg (PBW) and decreased daily (day 2: 7 ml/kg, day 3: 6.8 ml/kg, day 4: 6.5 ml/kg, day 5: 6.2 ml/kg, day 6: 6.0 ml/kg).

**Table 1:** Baseline characteristics of 93 patients who survived more than 6 days of mechanical ventilation for ALI

	All Patients (n = 93)	Survivors (n = 60)	Non-Survivors (n = 33)	*P *value (S vs. NS)
Age (Mean (SD))	46.4 (16.5)	42.0 (13.3)	54.3 (18.8)	<0.001
SAPS II	41.9 (14.2)	37.9 (14.2)	49.1 (11.0)	<0.001
APACHE II score	18.7 (7.1)	17.0 (7.3)	21.5 (5.85)	<0.01
LISS	2.65 (0.50)	2.62 (.05)	2.71 (0.5)	0.46
Lowest MAP	61.6 (11.4)	62.4 (11.5)	59.9 (11.3)	0.35
PaO2	105.4 (47)	105.4 (45.6)	105.2 (50.2)	0.98
PaCO2	43.2 (9.6)	42.7 (7.6)	44.1 (12.4)	0.48
FiO2	0.76 (0.24)	0.74 (0.25)	0.80 (0.21)	0.27
A-a Gradient	363 (182)	343.5 (191.3)	398.3 (160.3)	0.17
PaO2/FiO2	147 (60)	151.3 (58.8)	138.4 (62.3)	0.34
OI (cmH20/mm Hg)	11.4 (8.0)	10.0 (5.8)	13.9 (10.45)	0.02
Respiratory system compliance (ml/cm H20)	27.8 (10.4)	27.6 (8.8)	28.1 (13.0)	0.84
Tidal Volume (ml)	474.0 (125.6)	490.8 (116.3)	442.6 (137.8)	0.08
Tidal Volume (ml/kg PBW)	7.4 (2.2)	7.8 (1.9)	6.7 (2.5)	0.02
Duration of Mechanical Ventilation	21.4 (25.1)	20.5 (14.9)	23.1 (37.4)	0.64
pH	7.33 (0.10)	7.34 (0.09)	7.31 (0.11)	0.16
Base Deficit	-3.34 (5.98)	-2.94 (5.94)	-4.05 (6.08)	0.40
Gender (% female)	32%	29%	36%	0.63
Primary cause of ARDs	41%	43%	36%	0.65
Smoker	29%	32%	24%	0.48
Steroid use	17%	13%	24%	0.25
Trauma	8%	12%	0%	<0.05
Cirrhosis	11%	8%	15%	0.32
COPD	10%	8%	12%	0.72
Vasopressor	46%	35%	64%	0.01

*Definitions of abbreviations: *MAP, mean arterial pressure; PaO_2_/FiO_2_, ratio of arterial oxygen partial pressure-to-inspired oxygen fraction; PaO_2_, arterial oxygen partial pressure; PaCO_2_, arterial carbon dioxide partial pressure; FiO_2_, inspired oxygen fraction; COPD, chronic obstructive pulmonary disease. Data presented as mean (standard deviation) or percentage of total patients in each column. These data were collected on the day of study enrollment.

### Bivariate Analysis - Admission

There were several notable differences between survivors and non-survivors at the time of ALI diagnosis (Table 1). Non-survivors were older and had higher SAPS II and APACHE II scores. In addition, survivors were more likely to have trauma as the cause of their ALI (12% vs. 0%, p = 0.048) and had higher body weight corrected tidal volumes (7.8 ± 1.9 vs. 6.7 ± 2.5, p = 0.02). A higher percentage of non-survivors were on vasopressors at the time of ALI diagnosis (64% vs. 35%, p = 0.01), which may be due to a higher prevalence of septic shock in non-survivors. There was no difference in the percent of patients with a primary cause for ALI between survivors and non-survivors (primary cause in 43% vs. 36%, p = 0.65).

On the first day of lung injury, the OI was the only pulmonary variable that was predictive of death in bivariate analysis (Table 2). The average OI was 10 ± 6 cm H_2_O/mmHg in survivors and 13.9 ± 11 cm H_2_O/mmHg in non-survivors (p = 0.02). Other common measures of oxygenation, including PaO_2_/FiO_2 _and FiO_2 _were not predictive of death. Similarly, neither respiratory system compliance nor plateau airway pressure discriminated between survivors and non-survivors on the first day of ALI.

**Table 2:** Bivariate analysis of respiratory variables for 93 patients with acute lung injury who were ventilated for more than 6 days

Variable		Day 1	Day 2	Day 3	Day 4	Day 5	Day 6
**Crs (ml/cm H**_**2**_**0)**	S	27.6 (8.8)	28.1 (12.3)	29.0(11.1)	28.4 (12.0)	28.5 (10.7)	29.1 (10.5)
	NS	28.1 (13.0)	25.7 (10.3)	26.6 (12.1)	25.5 (9.6)	25.0 (8.9)	23.4 (8.8)
	p	0.80	0.35	0.35	0.25	0.13	0.01
**Pplat (cm H**_**2**_**0)**	S	26.4 (6.1)	26.5 (7.0)	26.0 (6.7)	25.6 (5.3)	24.9 (5.1)	23.9 (4.7)
	NS	26.9 (9.0)	28.5 (8.9)	27.6 (8.3)	27.1 (8.2)	26.6 (6.8)	28.4 (7.5)
	p	0.74	0.24	0.33	0.31	0.20	<0.01
**MV (L/min)**	S	10.3 (2.8)	9.7 (2.5)	10.2 (2.6)	9.8 (2.8)	9.9 (3.1)	9.5 (2.7)
	NS	9.7 (3.9)	10.1 (3.9)	11.1 (4.7)	11.8 (5.9)	10.9 (4.3)	10.5 (3.2)
	p	0.43	0.59	0.21	0.03	0.20	0.10
**FiO**_**2**_	S	0.7 (0.2)	0.6 (0.2)	0.6 (0.2)	0.6 (0.2)	0.5 (0.1)	0.5 (0.1)
	NS	0.8 (0.2)	0.6 (0.2)	0.6 (0.2)	0.6 (0.2)	0.6 (0.2)	0.6 (0.2)
	p	0.11	0.46	0.25	0.22	0.27	0.02
**OI (cmH**_**2**_**0/mm Hg)**	S	10 (5.8)	12.4 (7.9)	11.9 (7.1)	10.8 (7.1)	11.0 (8.6)	9.6 (5.7)
	NS	13.9 (10.5)	13.3 (9.8)	13.8 (8.6)	14.2 (8.8)	14.0 (9.8)	14.8 (12.7)
	p	0.02	0.65	0.28	0.06	0.17	0.02
**P/F ratio (cm H**_**2**_**0)**	S	120.2 (58.1)	133.2 (69.7)	141.7 (59.0)	151.0 (331.2)	165.0 (85.1)	170.4 (87.4)
	NS	106 (60.8)	119.1 (45.1)	135.9 (61.7)	132.9 (61.9)	129.2 (77.3)	138.1 (65.1)
	p	0.21	0.22	0.69	0.18	0.02	0.03
**PEEP (cm H**_**2**_**0)**	S	7.5 (2.9)	8.9 (3.0)	9.4 (3.0)	9.2 (3.0)	9.0 (3.2)	8.6 (3.2)
	NS	8.0 (3.2)	9.6 (3.4)	9.3 (3.6)	9.1 (3.6)	9.4 (3.8)	9.9 (3.8)
	p	0.45	0.30	0.90	0.89	0.65	0.09
**Paw (cm H**_**2**_**0)**	S	14.8 (4.1)	15.7 (4.9)	16.4 (4.0)	15.6 (3.9)	15.3 (4.2)	14.9 (4.3)
	NS	15.6 (5.9)	16.6 (5.6)	16.7 (5.8)	16.8 (5.9)	16.3 (5.1)	17.1 (5.4)
	p	0.47	0.41	0.77	0.26	0.32	0.03
**pH**	S	7.36 (0.1)	7.40 (0.1)	7.39 (0.05)	7.38 (0.06)	7.39 (0.06)	7.40 (0.05)
	NS	7.32 (0.1)	7.40 (0.1)	7.39 (0.05)	7.36 (0.07)	7.38 (0.07)	7.34 (0.06)
	p	0.11	0.77	0.87	0.05	0.32	<0.01
**Base Deficit**	S	-2.9 (6.0)	-2.2 (5.1)	-1.0 (4.9)	-0.3 (4.2)	0.6 (4.6)	1.6 (4.6)
	NS	-3.6 (6.4)	-2.3 (4.2)	-1.5 (4.0)	-2.6 (5.1)	-1.6 (5.0)	-1.5 (5.7)
	p	0.63	0.90	0.58	0.03	0.06	0.01
**Vt (ml/kg)**	S	7.8 (1.7)	6.6 (1.8)	6.4 (1.4)	6.3 (1.5)	6.2 (1.4)	6.2 (1.3)
	NS	6.7 (2.1)	6.4 (1.8)	6.5 (1.8)	6.6 (2.5)	6.0 (1.8)	6.0 (1.4)
	p	0.02	0.65	0.67	0.42	0.51	0.44

*Definitions of abbreviations: *BD, Base deficit; Crs, respiratory system compliance; FiO_2_, inspired oxygen fraction; MV, minute ventilation; OI, oxygenation index; PaO_2_, arterial oxygen partial pressure; PaO_2_/FiO_2_, ratio of arterial oxygen partial pressure-to-inspired oxygen fraction; PEEP, positive end-expiratory pressure; mean Paw, Mean airway pressure; P_plat_, end-inspiratory plateau pressure; V_T_, Tidal Volume; S, survivors; NS, nonsurvivors. Data presented as mean (standard deviation).

### Bivariate Analysis - Changes Over Time

**Figure 1:**
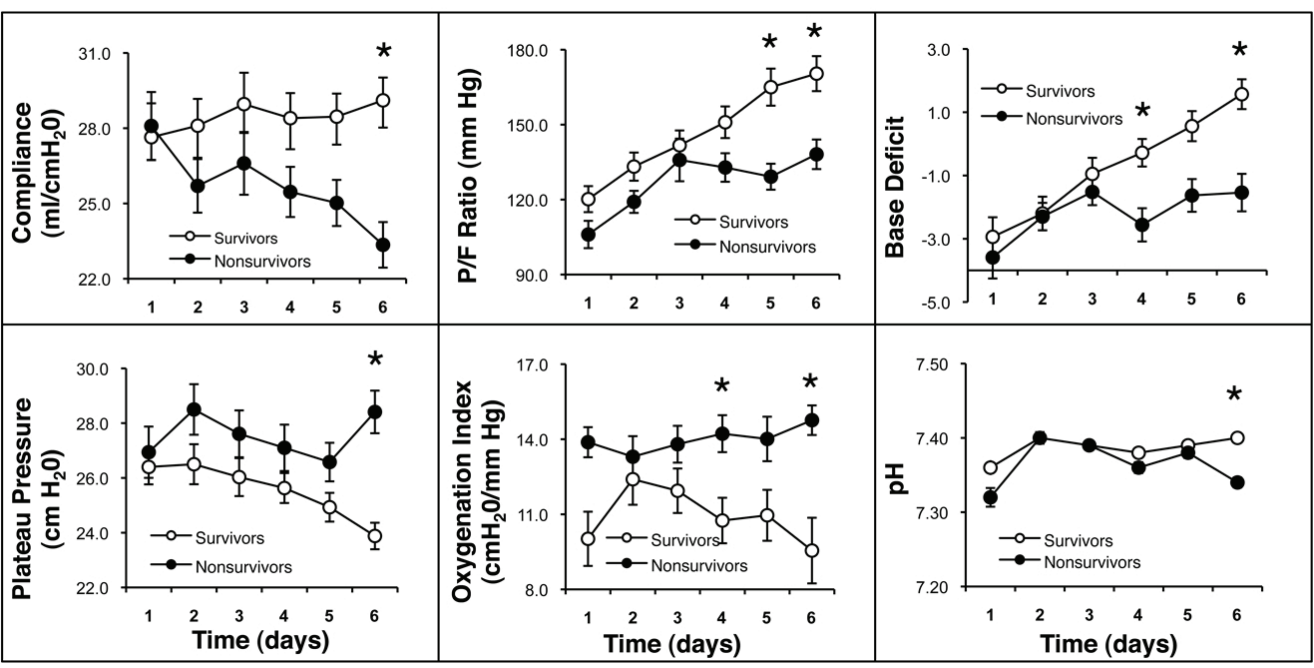
**Trends in measures of oxygenation, respiratory compliance and acid base balance during the first 6 days of mechanical ventilation for acute lung injury**. Data are shown as mean ± SEM. The * indicates p < 0.05.

On days two and three of ALI, none of the measured variables discriminated between survivors and non-survivors (Figure 1, Table 2). However, on days four, five and six of ALI, several measures of oxygenation, respiratory mechanics, and acid-base balance diverged, and the difference between survivors and non-survivors was statistically significant (Figure 1, Table 2). Specifically, on day 4, the , pH, and base deficit (BD), were predictors of death. On day 5, the PaO_2_/FiO_2 _was predictive of death, and by day 6, Respiratory system compliance, P_plat_, PaO_2_/FiO_2_, OI, mean Paw, pH, and BD were all predictive of death in the bivariate analysis.

### Multivariate Analysis

To identify variables that were independently associated with death, multivariate analyses were performed on variables that were associated with death in the exploratory bivariate analyses or of *a priori *interest. These variables included: OI, respiratory system compliance, BD, PaO_2_/FiO_2_, age, gender, COPD, pneumonia, vasopressors, APACHEII. In the multivariate analyses (Table 3), as in the bivariate analyses, OI was the only variable associated with death on the first day of lung injury (OR 2.16, p < 0.01). On day 6 of lung injury, the odds ratio for OI was elevated but did not reach statistical significance (OR 1.75, p = 0.09), however, PaO_2_/FiO_2 _(OR 2.09, p = 0.04) and respiratory system compliance (Crs) (OR 3.61, P = 0.01) were independently associated with mortality. In addition, odds ratios for the change between day 1 and day 6 of mechanical ventilation were calculated for each variable (Table 3). The only variable associated with death in these analyses was a decrease in the Crs (OR 2.14, p = 0.02) between days 1 and 6 of mechanical ventilation for ALI. Notably, the odds ratio for death for the decrease in Crs between days 1 and 6 was lower than the odds ratio for the absolute value of Crs on day 6.

**Table 3:** Multivariate adjusted odds ratio of death for selected variables on day 1, day 6 and the change in each variable between day 1 and day 6

		Day 1			Day 6		Δ Day 1 ➔ Day 6		
**Variable**	OR*	**95% CI**	**P**	OR*	**95% CI**	**P**	OR*	**95% CI**	**P**

PaO_2_/FiO_2_	1.02	0.55-1.87	0.96	2.09	1.05-4.15	0.04	1.71	0.94-3.12	0.08
OI	2.16	1.24-3.76	0.01	1.75	0.91-3.36	0.09	1.28	0.74-2.23	0.37
Crs	0.88	0.51-1.51	0.64	3.61	1.32-9.84	0.01	2.14	1.12-4.11	0.02
BD	0.77	0.41-1.43	0.4	1.45	0.77-2.72	0.25	1.72	0.94-3.15	0.08

* ORs of death are per standard deviation (SD) decrease for PaO_2_/FiO_2_, Crs, or BD and per standard deviation increase for OI.

*Definition of abbreviations: *OI, oxygenation index; Crs, quasistatic respiratory system compliance; BD, base deficit; OR, odds ratio; CI, confidence interval; p values <0.05 considered significant. *Description of model*: Stepwise, backward, multivariate logistic regression, p-values <0.1 remained in model and included: age, gender, COPD, pneumonia, vasopressors, APACHEII, worse pH on day 1. The main variables (PaO_2_/FiO_2_, OI, Crs, BD) were maintained in the final model regardless of p-value.

## Discussion

Predictors of death in established ALI are important and clinically relevant for two reasons. First, previous studies have reported an average duration of mechanical ventilation in ALI between 5 and 16 days, suggesting that a large proportion of patients with ALI are alive and mechanically ventilated 6 days after the diagnosis of ALI [13-15]. Second, important management decisions to escalate or limit the intensity of care are often made during this time interval. In this prospective cohort study of 93 patients with ALI who survived 6 or more days of mechanical ventilation, we found that a low or decreasing respiratory system compliance on the 6th day of mechanical ventilation was associated with an increased risk of death. This finding is novel because few other studies have identified pulmonary predictors of mortality in ALI patients ventilated with lung-protective ventilation during this stage of disease. In addition, if prospectively validated, these findings may help identify patients who are failing traditional therapy and who might benefit from novel rescue therapies.

Although the cumulative risk of complications associated with mechanical ventilation, including ventilator-associated pneumonia and sepsis, increases with each ventilator day, we were surprised to find that the mortality of patients who were ventilated for 6 or more days was similar to patients enrolled in our previous study [4] which included patients who died or were extubated during the first 6 days of ALI (35% vs. 42%, p = 0.42). Other studies, including the Kings County Lung Injury Project and the ARDSNet trial of steroids for persistent ARDS, reported surprisingly low mortality rates in patients requiring prolonged ventilation for ALI as well [13,14,16,17]. The low mortality in this study may be because the sickest patients who present with severe shock, catastrophic injury, or refractory hypoxemia die during the first 6 days of ALI and thus were not included in this analysis.

Previous studies of pulmonary predictors of mortality in ALI have focused on early predictors of mortality [18,4,20] or on physiologic changes between the onset of ALI and the third day of mechanical ventilation [21,19,20] in patients ventilated with traditional tidal volumes. In this study, the OI was the only variable predictive of death on the first day of lung injury in bivariate analysis. The OI was an independent predictor of mortality in the complete cohort of patients [4], and as previously published, is a clinically practical, early predictor of death in both adult [4] and pediatric [22] ALI populations. Contrary to previous reports [3], measures of pulmonary mechanics, including respiratory system compliance and P_plat_, were not predictive of death on the first day of mechanical ventilation. This difference from prior studies may be partially attributable to the use of lung-protective ventilation, which could attenuate alveolar stretch during mechanical ventilation.

On the 2^nd ^and 3^rd ^day of ALI, none of the physiologic variables measured in this study were associated with death. In contrast, previous studies found one or more predictors of death on days 2 and 3 of ALI. Cooke *at al *[19] examined predictors of mortality in a cohort of 1,113 patients with ALI and found that the change in PaO_2_/FiO_2 _ratio between the day of diagnosis and day 3 of hospitalization was predictive of death. Similarly, Estenssoro *et al *[20] examined a cohort of 217 patients in Argentina and found that the PaO_2_/FiO_2 _ratio was predictive of death on the third day of mechanical ventilation. Lastly, Gajic *et al *[18] retrospectively analyzed multiple physiologic variables on day 3 of mechanical ventilation in a large observational trial and then validated it in two independently collected data sets. Gajic *et al *found that PaO_2_/FiO_2 _ratio, P_plat_, mean Paw, PEEP and OI on the third day as well as the change in OI and PEEP between days 1 and 3 were predictive of a composite end point of death or ventilator dependence 15 days after intubation.

Three important distinctions may account for the differences between our study, which did not identify pulmonary predictors of death on days 2 or 3 of ALI and the 3 studies reported above. First, patients in our study were managed with a lung-protective ventilation strategy, which may standardize plateau airway pressure and oxygenation. Second, the cohort size may have limited our ability to find statistical differences between survivors and non-survivors on days 2 and 3 of mechanical ventilation. Lastly, our study included only patients who survived >6 days of ALI, thus eliminating patients who died early due to refractory hypoxemia, catastrophic trauma or fulminant septic shock. Although the subgroup of patients who die of hypoxemia is small (approximately 15%), this difference could have driven the statistical significance of the PaO_2_/FiO_2 _ratio on the third day of ALI in prior studies [23,24].

The major finding of this study was that a low or decreasing respiratory system compliance on the 6^th ^day of mechanical ventilation is an independent predictor of mortality in this cohort of patients. Respiratory system compliance may decrease in non-survivors due to a combination several factors, including volume overload, atelectasis and early pulmonary fibroproliferation. Patients with refractory shock may have required more fluid boluses to maintain adequate blood pressures and this may have lead to worsening pulmonary and chest wall edema. Although our data set had greater than 15% missing data for volume administration, there was no statistical difference in daily or cumulative fluid balance between survivors and non-survivors. A higher level of lung collapse and atelectasis may also contribute to decreased compliance in non-survivors. The amount of recruitable lung identified by CT scanning has been shown by others to be associated with mortality during ALI [25]. In the context of our data, lower respiratory system compliance may be indicative of more atelectasis and thus relative over-distention of healthier lung units despite the use of lung-protective ventilation. Lastly, respiratory system compliance may decrease in non-survivors due to the fibroproliferative phase of ALI which can occur as early as the 6^th ^day of mechanical ventilation in autopsy studies [26]. Biochemical studies have identified procollagen peptide I and III, which are precursors of fibrotic collagen, in BAL fluid at the time of ALI diagnosis and the amount of peptide in this specimens correlates with mortality [27]. Future studies utilizing esophageal manometry to accurately estimate the contribution of chest wall or abdominal pressure to total respiratory system compliance, with more complete records of volume administration and weight changes as well as pathologic studies of patient who die during the later phases of ALI could provide a mechanistic explanation for these physiologic findings.

This study has several limitations. First, the small study size may have limited our ability to detect statistical differences in physiologic variables on days 2 and 3 of mechanical ventilation. Second, this study was conducted at an academic and county hospital; thus these findings might not be generalizable to community hospital populations. Third, *post-hoc *selection of patients can lead to selection bias; however, we believe that our strict criterion (>6 days of mechanical ventilation) for entry into this study was the best way to answer our study question. Fourth, extensive information on transfusion of blood products, a known risk factor for ALI, were not collected. Lastly, due to a small study size we were unable to divide this population into a derivation and validation cohort. Future replication of these findings in a separate cohort of patients with ALI would substantiate our results.

## Conclusions

In conclusion, we studied the association of changes in pulmonary physiologic variables with death in a cohort of ALI patients who were mechanically ventilated for more than 6 days. Using multivariate analysis, we found that both the absolute value of respiratory system compliance on day 6 and the decrease in respiratory system compliance between days 1 and 6 of mechanical ventilation for ALI are associated with increased mortality. We hypothesize that decreased respiratory system compliance may be indicative of persistent pulmonary or chest wall edema, atelectasis of inflamed lung or evidence of the early fibroproliferative phase of ALI. If these results can be replicated prospectively in a larger set of ALI patients, then a low or decreasing respiratory system compliance, interpreted in the context of other known predictors of mortality in ICU patients, may help identify patients at the highest risk of death from ALI during the subacute phase of illness.

## Competing interests

The authors declare that they have no competing interests.

## Authors' contributions

RK, DM and MM generated the original idea for this research study and collected the data. ES collated, analyzed and interpreted the data. ES created the figures and wrote the manuscript. HJ and ME helped with the statistical analysis. RK and MAM oversaw the research, helped analyzed the data and edit the manuscript. All authors have read and have approved this manuscript.
